# Scientific frontiers on migration and sustainability

**DOI:** 10.1073/pnas.2321325121

**Published:** 2024-01-08

**Authors:** William Neil Adger, Sonja Fransen, Ricardo Safra de Campos, William C. Clark

**Affiliations:** ^a^Department of Geography, Faculty of Environment, Science and Economy, University of Exeter, Exeter EX4 4RJ, United Kingdom; ^b^United Nations University–Maastricht Economic and Social Research Institute on Innovation and Technology, and School of Economics and Business, Maastricht University, Maastricht 6211 AX, The Netherlands; ^c^Global Systems Institute, Department of Geography, Faculty of Environment, Science and Economy, University of Exeter, Exeter EX4 4RJ, United Kingdom; ^d^Sustainability Science Program, Harvard Kennedy School of Government, Harvard University, Cambridge, MA 02138

The great acceleration of economic activity and environmental degradation globally over the past decades is creating unprecedented challenges to the goals of sustainable development. In parallel, the redistribution of population through migration has created under-appreciated dynamics involving changes in human well-being, resource pressures, innovation and adaptation, and governance. The PNAS Special Feature to which the present paper serves as an Introduction explores the implications of migration for sustainable development.

The challenge of sustainable development is to guide the interactions between nature and society so that they advance the well-being of people in the here and now without undermining the ability of people elsewhere or in the future to advance their own visions of a better life (1). The principal ways that migration can alter those interactions include changing demands for renewable and non-renewable resources and other environmental burdens in origin and destination areas; changing demographic structures (such as aging populations, increased dependency ratios, and average household size) and their consequences for environmental burdens; and changing fiscal and human capital movements with their consequences for innovation (remittance flows, brain gain, and brain drain) and adaptation more generally. In parallel, migration flows themselves are affected by global environmental change, altering existing flows and creating new flows, not least of involuntary and distress migration in the face of environmental degradation and climate extremes. All of these trends and hidden phenomena are filtered through the real-world politics of migration, which makes migration a highly contested and emotive topic.

As context for making sense of ongoing research on migration and sustainability, including the work presented in this Special Feature, some basic definitions and numbers are helpful.

Migration, as commonly used across social science and in government statistical offices, is the long-term movement or flow of people from a place of residence across an administrative boundary. Migrants are the stock of people who have made such movements. How many migrants are there? How far have they migrated? Why did they move? Answers to such questions are time and context dependent, and subject to debates about definitions and data reliability. But the global snapshot for the year 2020, reproduced in Table 1 using data from the *World Migration Report* (WMR) (2), is a useful starting point. It shows that in that year, about 15% of the world’s people were migrants, i.e., residing a significant distance from their place of birth. The majority of these—perhaps 10% of the total population—were voluntary internal migrants, i.e., had moved by choice within their countries of birth, generally in pursuit of better opportunities to advance their well-being. Just 1% were involuntary or “displaced” migrants, with most of those fleeing social conflict to places that were still within their original countries. Migration as a response to environmental stress involved only about 0.1% of the world’s population, which nonetheless meant that more than seven million of the planet’s people in 2020 were living in places far from their homes, displaced by environmental disasters. Historical data suggest that the 2020 snapshot would have looked about the same in terms of proportion of the world’s (growing) population for most of the preceding decades. Dramatic increases in wars and climate-related shocks since 2020, however, mean that a snapshot today would almost certainly present a grimmer global picture of both the number and proportion of displaced migrants. What remains true, however, is that while displacement of people in response to environmental pressures is a very small part of the global migration story, for the places and people that are directly impacted in environmental disasters, migration and migrants is a very big deal indeed. As climate change and other environmental stresses intensify in coming years, more people will almost certainly be displaced and more places will almost certainly be transformed by migration. It is to the contexts in which environmental disasters, involuntary migration, and the pursuit of sustainability intersect that we now turn.

**Table 1.** Global migrant stocks: Snapshot for 2020^*^

Numbers (millions)	Internal	International	Total
Regular (voluntary)	~740 M^†^	281 M (*169 M migrant workers*)^‡^	~1,021 M
Displaced (involuntary)^§^	55 M (*48 M by conflict*) (*>7 M by disasters*¶)	34 M (*26 M refugees*) (*8 M other*^#^)	>89 M
Total	**>**795 M	311 M	**>**1,106 M
% All migrants			
Regular (voluntary)	67%	25% *(15% migrant workers)*	92%
Displaced (involuntary)	5% (*4% by conflict*) (*1% by disasters*)	3% (*2% refugees*) (*1% other*)	8%
Total	72%	28%	100%
% World population^||^			
Regular (voluntary)	9.5%	3.6% (*2.2% migrant workers*)	13.1%
Displaced (involuntary)	0.7% (*0.6% conflict*) (*>0.1% disasters*)	0.4% (*0.3% refugees*) (*0.1% other*)	>1.1%
Total	>10.2%	4.0%	>14.2%

^*^Data are all drawn from WMR 2022 (2), which refers the reader to the many original sources it has drawn from for caveats and assumptions. Except where noted, data are meant to provide a snapshot for the year 2020. Disruptions since then from the COVID-19 pandemic, multiple new wars and intensifying environmental calamities mean that a snapshot today or in the future would likely be different in many ways.

^†^These data are for 2009, which WMR 2022, citing UN sources, calls the “most recent available” of a quantify difficult to estimate accurately because of a variety of conceptual and measurement issues which it discusses.

^‡^Numbers in (parentheses) and *italics* are subsets of the numbers immediately above.

^§^For involuntary displaced people who remain internal to their home countries, WMR distinguishes whether their displacement can be primarily attributed to conflict and other forms of violence or to environmental disasters, which mostly involve water. For people who are displaced across national borders, WMR 2022 classifies them as “refugees” independent of the primary cause of their displacement. It notes, however, that most persons displaced by primarily disasters have so far remained internal to their own country.

^¶^WMR refers to the number of people internally displaced by disasters as a significant underestimate because data limitations mean that includes only people displaced in 2020, not those still displaced from earlier disasters. Updates will aim to include in this category people still displaced from all disasters.

^#^”Other” includes asylum seekers plus additional Venezuelans classified as neither refugees or asylum seekers.

^||^World population in 2020 was 7,821 M people.

Cross-disciplinary research over the past decade has begun to provide coherent evidence of the scope and scale of migration-environment linkages in all these areas and to demonstrate how migration and movement in general has the potential to act as a stimulus to sustainability. Some of this evidence comes from large international assessments, including within the Intergovernmental Panel on Climate Change (3), the UK Foresight report of the UK Office for Science on Migration and Global Environmental Change (4), as well as international collaborative assessments (5). The field has been dominated by a focus on how environmental change drives flows of migration and mobility, sometimes in ways that are argued to be over-deterministic and not reflecting historical contexts (6). Hence, this Special Feature highlights insights at the frontiers of these areas to interrogate how sustainability science can better incorporate migration research when appraising the range of risks, dynamics, and opportunities for societal transformation (7).

Migration associated with environmental change is already happening. Environmental change is not the main driving force of current migration patterns, even in the most exposed places. But it is a major driver of movement for particular places and communities and represents a matter of restricted choice and ultimately of environmental justice. At the larger scale, climate change is likely to become a more important driver of migration over time, through changes in economic opportunities and the relative attractiveness of places of origin and destination—often referred to as push and pull factors of migration (8). But the figure of the environmental migrant is elusive and almost always politicized, not least in being raised as a threat to social order and insecurity. Baldwin (9) argues that this security orientation is inherently racialized, not least because it invokes migration by default as international movement and implies cross-cultural social tensions.

Migration is deeply historically rooted in relations between global regions, often in exploitative and colonial relations and past injustices. The movement of capital to find new markets, often through imperialist expansion, is a profound driver of increased movement of people within and across countries. Multi-cultural societies are products of recent and historic flows of people and all cultures are outcomes of such flows. The role of cultural integration has been analyzed as a set of acculturation and convergence. Immigration leads to increased ethnic diversity in destination societies; a process which has both short- and long-term consequences for social cohesion and social trust. As such, the successful integration of new migrant populations is essential for the successful pursuit of sustainability in host societies.

The papers in this Special Feature are based on cross-disciplinary analysis across the demographic, social, and environmental sciences. They draw on observations and modeling across geographies ranging from coastal US states, to refugee camps in Asia and Africa, and movements across Pacific islands and agriculture-dominated economies. Together, they highlight an emerging sustainability science focus on both systematic interactions between causes and consequences of migration and a nuanced view of the role of ongoing migration in shaping trajectories and pathways toward sustainability.

The scope of the papers in this Special Feature encompasses the role of environmental risks on involuntary movement of people, and the outcomes of those migration flows. This includes vulnerability of populations to climate risks in refugee camps, trends in displacement from flood risk amplified by local context and governance, and the consequences of populations moving as a result of sea level rise resulting in remnant aging populations. Each of these studies highlights that environmental degradation makes dealing with these displaced and trapped populations doubly difficult. The papers encompass systems approaches to migration as multi-sited lives and livelihoods and the role of place and belonging in dampening likely outward migrations from places at risk. At scales from sub-national to international, there is therefore potential for interventions and insights that align governance of migration and governance of resources to promote sustainability.

The scope and nature of the migration-sustainability relationship is described in an overview Perspective paper for the Special Feature written by Adger et al. (1): Migration is part of a complex adaptive system, influenced by and influencing many nature–society interactions. They outline that the outcome of migration in both origin and destination regions involves interactions between processes of changing well-being, governance, innovation, and adaptation. These immediate processes are shaped by prior demographic transitions, the demand for labor, and by political barriers and regulation of movement. In normative terms, migration will only promote sustainable development when the outcomes of environmental burdens and resource use are themselves sustainable under new demographic realities. The Perspective paper highlights, in line with other migration policy analysis (10), that distinctive policies to promote sustainability are needed for each of the principal flows: regular migration dominated by economic motives and involuntary displacements due to conflicts or disasters.

Migration has impacts on the dynamics of sustainability through changing the demography of places. Hauer et al. (11) show that increased flood risk in areas of Florida shifts populations to safer destinations. And since climate migration is most likely to occur among working-age adults, the places at risk from sea level rise will also in tandem experience more rapidly aging populations. Realistic scenarios of sea level change and population densities for the coastal United States show that perhaps 1.5 million people could be directly displaced under central scenarios. But those most likely to leave are working age and hence promote economic activity and further population growth in destination areas. Hence, there is a so-called demographic amplification that may be up to 15 million—ten times the direct effect—due to those places with growing populations being more attractive and attracting other investment and people. This study resonates with findings elsewhere on sea level changes and their consequences for migration (12), with all studies suggesting that adaptations in place can reduce pressures on outward movement. But surging population change due to the demographic amplification from actual and anticipated sea level change, as identified by Hauer, leads to sustainability challenges for both origin and destination areas. Aging population structures reduce the tax base for public investment in shrinking populations, while the continued growth of destination areas poses familiar dilemmas for resource scarcity and management of water, air quality, and urban expansion.

Migration associated with environmental risks is amplified by social and political context. Vestby et al. (13) examine key factors that contribute to flood-induced displacement using georeferenced floods worldwide, 2000 to 2018. Their approach focuses on socioeconomic, political, and security contexts to assess the extent to which these societal conditions mediate flood-induced displacement. Their findings suggest that socioeconomic factors, including poverty and inequality at country level, amplify the impact of floods on vulnerable populations. Poorer communities in developing countries often lack resources to prepare for and recover from floods, making them more likely to be displaced. Informal settlements in flood-prone areas, characterized by substandard housing and inadequate infrastructure, are particularly at risk. Conversely, effective governance and disaster management systems are crucial in mitigating the impact of floods and reducing displacement. Their predictive models for world regions including Europe, Americas, Africa, and Asia and Oceania suggest that poor governance, conflict, or inadequate response mechanisms can magnify displacement and hinder recovery efforts. Inclusive democracy and peace are important indicators to overcome the impacts of disasters and enhance sustainability for exposed societies.

Involuntary migrants themselves are also susceptible to environmental risks. Fransen et al. (14) study the extent to which the 20 largest refugee settlements worldwide are exposed to extreme weather events, and whether this exposure deviates from the average national weather conditions in the settlements’ host countries. They show that the majority of refugee settlements are highly exposed to slow-onset stresses. These stresses include elevated temperatures (in settlements located in Kenya, Ethiopia, Rwanda, Sudan, and Uganda), colder temperatures (in the case of Jordan and Pakistan), and low rainfall (in Ethiopia, Rwanda, Kenya, and Uganda). These exposure levels surpass the national averages in these respective countries. Refugee settlements in Bangladesh were found to be exposed to extreme rainfall. These findings confirm that refugee settlements are typically located in risky locations in their host countries, which may further increase the vulnerability and marginalization of refugee populations. These results suggest that countries hosting displaced populations need to include these in their climate adaptation planning and sustainable development policies, if equitable and sustainable development pathways are to be created in these countries. Furthermore, inclusive sustainable development policies are also essential for society’s commitment to the “leave-no-one behind” global policy agenda.

Sustainable development is more than what happens in single locations: Migration leads to multi-sited lives and livelihoods, the spreading of risk across time and space, and perhaps to more resilient systems. Sakdalporak et al. (15) examine various dimensions of such trans-local resilience in the context of migration as a response to environmental change. Specifically, they focus on the social–spatial differentiation of migration as adaptation, drawing on empirical data collected in Thailand. The concept of trans-local social resilience refers to the ability of communities to adapt and respond to environmental challenges through social connections and networks that extend beyond their immediate locality. It emphasizes the intricate relationship between local embeddedness, the deep integration and involvement in a particular local community or geographic location, and trans-local connectedness, i.e., the connections and interactions that extend beyond the local context. These are manifest in the form of directed, adaptative actions in agricultural livelihoods at the site of origin, for example, enabling intensification and the change to cash crops, as well as the reduction of vulnerability, by increasing and diversifying income, and by improving households’ asset base over time, including investment in education of children. This study demonstrates the challenges and opportunities associated with trans-local migration and its implications for both places of origin and destination.

Sustainability is also manifest in values and desires that shape both adaptation practices and people’s decisions to remain in place. Jarillo and Barnett (16) focus on the concept of belonging in sustainability pathways and argue that a sense of belonging is essential for migration to contribute to sustainability outcomes for origin communities. They study the interactions between out-migration, belonging, and climate adaptation in three atoll islands in the Pacific, which are heavily affected by climate change. The findings illustrate how a sense of belonging encourages migrant communities to engage financially with their home communities, a process that is facilitated by the presence of strong social capital. Belonging is what connects communities and migrants across different locations and time, and it is this sense of belonging that allows migration to play an essential role in adaptation processes within sustainability transitions on the islands. The economic opportunities that migrants have in their destination areas are an important facilitating factor as well as the availability of communication infrastructure. This study contributes to sustainability science by highlighting the essential role of belonging in migration and its impact on climate adaptation and pathways of sustainable development.

Sustainable development requires foresight about the consequences of choices affecting population, affluence, and technology. Most sustainability policy assumes, as Hauer puts it, that migration is simply a rearrangement of people across space. Yet such redistribution has major consequences for the sort of people who are within a political jurisdiction—both in terms of their economic, age, and demographic profile, but also in terms of values and perspectives. Yet the two arenas of politics and policy are rarely integrated. This is demonstrated by Zickgraf et al. (17) who show clearly the siloed nature of migration governance and environmental governance for specific countries. They find that national and regional policy makers tend to focus on the social dimensions of sustainability, multi-culturalism, and social cohesion for new migrant populations in their analysis of European and North American cities. Hence, the governance of water resources, land, and pollution simply takes migration as an exogenous factor in growing or declining population.

The same blind spots in sustainability governance are manifest on the international stage where international agreements on biodiversity and climate change perceive migration as a threat or consequence of environmental change, rather than an integral part of the transformation of nature–society interactions in the modern world. In that light, international discussions around safe and regular international migration (the United Nations’ Global Compact for Safe, Orderly and Regular Migration), through to the Sustainable Development Goals, and discussions on adaptation to climate change (through the Green Climate Fund of the Framework Convention on Climate Change) relegate migration and the movement of populations to afterthoughts (18). Integration of migration into both sustainability science and the governance of sustainability require, according to Zickgraf, recognition of the social and demographic consequences of population movements. Migration potentially transforms economic activity and social and political institutions in both places of origin and destination—through the flow of ideas, material resources, and increased social ties between remote places. These in turn make governance necessarily focused on those linkages—on facilitating movement and return rather than discouraging it, and on cooperation between neighboring jurisdictions for the mutual benefit of both

The papers in this Special Feature highlight that the pursuit of sustainability will alter the economic geography of every part of the world—where the economy makes products, engages in trade, and provides services. It will therefore alter the distribution of opportunities and employment and by that mechanism will alter settlement patterns and the movement of people to a place where sustainable development is possible. Yet we are not likely to perceive this change in the same manner as observing mass or sudden migration. The theories, systems approaches, and data highlighted in this Special Feature point the way to an integrated sustainability science for meeting this challenge.

## References

[1] W. N. Adger, S. Fransen, R. Safra de Campos, W. C. Clark, Migration and sustainable development. Proc. Natl. Acad. Sci. U.S.A. 121, e2206193121 (2024). 10.1073/pnas.2206193121 PMC10801908 38190541

[2] M. McAuliffe, A. Triandafyllidou, Eds., “World migration report 2022” (International Organization for Migration, Geneva, 2021).

[3] G. Cissé, R. McLeman, H. Adams, P. Aldunce, “Health, wellbeing, and the changing structure of communities” in Climate Change 2022: Impacts, Adaptation and Vulnerability. Contribution of Working Group II to the Sixth Assessment Report of the Intergovernmental Panel on Climate Change. H.-O. Pörtner, D.C. Roberts, M. Tignor, et al. Eds. (Cambridge University Press, 2022). pp. 1041–1170.

[4] The Government Office for Science, “Foresight: Migration and global environmental change final project report” (The Government Office for Science, London, 2011).

[5] D. Ionesco, D. Mokhnacheva, F. Gemenne, The Atlas of Environmental Migration (Taylor and Francis, 2016).

[6] R. M. Horton, A. de Sherbinin, D. Wrathall, M. Oppenheimer, Assessing human habitability and migration. Science **372**, 1279–1283 (2021). 34140379 10.1126/science.abi8603

[7] W. C. Clark, A. G. Harley, Sustainability science: Toward a synthesis. Ann. Rev. Environ. Resour. **45**, 331–386 (2020).

[8] C. Cattaneo , Human migration in the era of climate change. Rev. Environ. Econ. Policy **13**, 189–206 (2019).

[9] A. Baldwin, Premediation and white affect: Climate change and migration in critical perspective. Trans. Ins. Br. Geograph. **41**, 78–90 (2016).

[10] S. Huckstep, M. Clemens, Climate change and migration: An omnibus overview for policymakers and development practitioners Policy Paper 292. (Center for Global Development, 2023).

[11] M. E. Hauer, S. Jacobs, S. A. Kulp, Climate migration amplifies demographic change and population ageing. Proc. Natl. Acad. Sci. U.S.A. 121, e2206192119 (2024). 10.1073/pnas.2206192119 PMC10801880 38190539

[12] L. Reimann , Exploring spatial feedbacks between adaptation policies and internal migration patterns due to sea-level rise. Nat. Commun. **14**, 2630 (2023). 37149629 10.1038/s41467-023-38278-y PMC10164174

[13] J. Vestby, S. Schutte, A. F. Tollefsen, H. Buhaug, Societal determinants of flood-induced displacement. Proc. Natl. Acad. Sci. U.S.A. 121, e2206188120 (2024). 10.1073/pnas.2206188120 PMC10801835 38190537

[14] S. Fransen, A. Hunns, M. Sirenko, T. Comes, Refugee settlements are highly exposed to extreme weather conditions. Proc. Natl. Acad. Sci. U.S.A. 121, e2206189120 (2024). 10.1073/pnas.2206189120 PMC10801877 37276435

[15] P. Sakdapolrak , Trans-local social resilience dimensions of migration as adaptation to environmental change. Proc. Natl. Acad. Sci. U.S.A. 121, e2206185120 (2024). 10.1073/pnas.2206185120 PMC10801836 38190538

[16] S. Jarillo, J. Barnett, Migration, belonging, and the sustainability of atoll islands through a changing climate. Proc. Natl. Acad. Sci. U.S.A. 121, e2206190120 (2024). 10.1073/pnas.2206190120 PMC10801890 38190530

[17] C. Zickgraf, D. Jolivet, C. Fry, E. Boyd, A. Fábos, Bridging and breaking silos: Transformational governance of the migration-sustainability nexus. Proc. Natl. Acad. Sci. U.S.A. 121, e2206184120 (2024). 10.1073/pnas.2206184120 PMC10801863 38190527

[18] C. Bryne, Climate change and human migration. UC Irvine L. Rev. **8**, 761–790 (2018).

